# Factors associated with delayed reporting for surgical care among patients with surgical acute abdomen attended at Muhimbili National Hospital: Tanzania

**DOI:** 10.1186/s12876-023-02659-w

**Published:** 2023-03-08

**Authors:** Maryam Hamdan, Xu Yang, M. Mavura, Mohammed Saleh, George Kannani, Kang Haonan, Abdullah Al-danakh, Xu Zhaohui, Gong Zezhong, Ri Hyokju, Boureima Amado, Ren Yanying, Chen Xin

**Affiliations:** 1 Department of Hernia and Colorectal Surgery, Second Hospital of Dalian Medical University, Dalian, China; 2 Department of General Surgery, Muhimbili National Hospital (MNH), Dar es Salaam, Tanzania; 3 Department allied, Health Science Zanzibar, Zanzibar, Tanzania; 4 Department of Urology, The First Affiliated Hospital of Dalian Medical University, Dalian, China

**Keywords:** Surgical acute abdomen, Demographic, Social factors, Health staff, Late presentation, Surgery, Muhimbili National Hospital

## Abstract

**Background:**

Surgical acute abdomen is a sudden onset of severe abdominal symptoms (pain, vomiting, constipation etc.) indicative of a possible life-threatening intra-abdominal pathology, with most cases requiring immediate surgical intervention. Most studies from developing countries have focused on complications related to delayed diagnosis of specific abdominal problems like intestinal obstruction or acute appendicitis and only a few studies have assessed factors related to the delay in patients with acute abdomen. This study focused on the time from the onset of a surgical acute abdomen to presentation to determine factors that led to delayed reporting among these patients at the Muhimbili National Hospital (MNH) and aimed to close the knowledge gap on the incidence, presentation, etiology, and death rates for acute abdomen in Tanzania.

**Methods:**

We conducted a descriptive cross-sectional study at MNH, Tanzania. Patients with a clinical diagnosis of the surgical acute abdomen were consecutively enrolled in the study over a period of 6 months and data on the onset of symptoms, time of presentation to the hospital, and events during the illness were collected.

**Results:**

Age was significantly associated with delayed hospital presentation, with older groups presenting later than younger ones. Informal education and being uneducated were factors contributing to delayed presentation, while educated groups presented early, albeit the difference was statistically insignificant (*p* = 0.121). Patients working in the government sector had the lowest percentage of delayed presentation compared to those in the private sector and self-employed individuals, however, the difference was statistically insignificant. Family and cohabiting individuals showed late presentation (*p* = 0.03). Deficiencies in health care staff on duty, unfamiliarity with the medical facilities, and low experience in dealing with emergency cases were associated with the factors for delayed surgical care among patients. Delays in the presentation to the hospital increased mortality and morbidity, especially among patients who needed emergency surgical care.

**Conclusion:**

Delayed reporting for surgical care among patients with surgical acute abdomen in underdeveloped countries like Tanzania is often not due to a single reason. The causes are distributed across several levels including the patient’s age and family, deficiency in medical staff on duty and lack of experience in dealing with emergency cases, educational level, working sectors, socioeconomic and sociocultural status of the country.

## Introduction

About 1% of hospital admissions are in cases of surgery for patients with a surgical acute abdomen, accounting for 5–10% of all visits to emergency departments [1, 2]. The majority of patients who suffer from severe abdominal pain are in relatively good health and in most cases, this pain can last for up to 6 h, requiring surgical treatment [2–4]. The pain can be felt in any part of the abdomen that may indicate a life-threatening intra-abdominal pathology requiring urgent surgical intervention. Most causes reside within the abdomen and are either caused by infections, inflammation, vascular occlusion, or obstruction. [5–7], however, there are a few exceptions too. Acute abdomen is a notoriously difficult symptom posing substantial challenges to surgeons due to its diagnostic ambiguity and risk of misinterpretation because it can be either visceral, somatic, or referred pain [6, 8]. Surgically, acute abdominal conditions have a range of diagnoses depending on the patient’s age and sex [8, 9]. Younger people are more likely to have acute appendicitis, whereas older patients are more likely to develop biliary diseases, large and small bowel obstruction, intestinal ischemia, perforated peptic ulcer diseases, and diverticulitis. [7, 10] According to the population and geographical region, acute appendicitis, non-specific abdominal pain, pain of urological origin, intestinal obstruction, and biliary tract diseases are the most common causes of abdominal pain requiring admission [7, 11, 12]. Delays in diagnosis and treatment raise the risk of death, morbidity, and psychological stress among these patients [7, 11]. A wide variety of factors can lead to acute abdomen, and the relative occurrence of these factors varies between populations. Clinicians consider multiple diagnoses seriously, particularly in situations where rapid intervention is necessary to reduce morbidity and mortality [9, 12, 13].

Previous studies have linked the following factors to delayed or inaccurate diagnoses of acute appendicitis in adults aged 60 years or more [10, 14]: atypical symptoms or inadequate examination, poor clinical findings, the level of experience of the emergency physician, female sex, the presence of coexisting conditions, constipation, and appendicitis without pain [11, 15, 16]. Other clinician-related and environmental variables, including the physician's specialty, availability for ultrasonography, and hospital size, have been described as factors related to misdiagnosis in pediatric research [16, 17].

The majority of patients in developing nations like Tanzania present late in the disease course, which may result in poor outcomes [10, 13, 18]. Surgeons often experience a substantial delay between the time onset of the disease to the time of treatment in patients with acute abdomen requiring surgery in Tanzania.

No study has previously investigated the variables leading to delayed presentation of the patient with acute abdomen requiring surgery in adults in Tanzania. Existing published work relied on emergency department physicians or nurses identifying a primary complaint, whereby the real determinants of the patients with surgical acute abdomen who present late in the ED and GSD cannot be established.

In this study, we aimed to analyze the period from the onset time of the disease to the time of treatment in patients with surgical acute abdomen in Muhimbili National Hospital (MNH) and identify factors leading to a delayed presentation.

We also assessed the mortality and morbidity associated with the delayed presentation in patients needing emergency surgical care.

## Methodology

### Study area

This was a 6-month long descriptive cross-sectional study (from March 2022 to August 2022), conducted in the Emergency and General Surgery department at MNH, a tertiary care center located in the Dar Es Salaam region, which is in the city of Tanzania. It is a National referral hospital with 500 beds and between 1000 and 1200 admissions per week [9]. MNH receives patients from all other peripheral hospitals in Tanzania and has a research center and university. All patients who presented to the ED and GSD with a clinical diagnosis of acute abdomen throughout the research period were enrolled in the study. The approval of the institutional ethics committee was obtained before the study and informed consent to participate in the study was provided by the patients before the interview. We excluded all patients who did not consent to participate in our study, patients receiving life-saving procedures such as CPR, insertion of a feeding tube, and mechanical breathing/ventilation were excluded. Those who were unconscious and intoxicated, or unable to be questioned owing to psychiatric disorders were excluded.

### Data collection

Data were collected by interviewing the patient, family member, doctor, or nurses by using a standard questionnaire performed in Swahili and English language either before surgery or postoperatively, depending on the patient’s status. The information included in the socio-demographic details comprised name, age, sex, marital status, occupation, education, etc. In an attempt to calculate the distance from the hospital, the complete residential address of the patient was recorded. We defined surgical acute abdomen as abdominal pain due to a surgical cause that necessitated admission to the surgical unit. The time of onset was defined as the period when the first abdominal symptom began, and the time of presentation was the time of admission to the hospital. The pre-hospital interval was the time difference between onset and presentation. Delayed presentation was defined as a pre-hospital interval of > 24 h. We enquired in detail about the events between the onset of symptoms and admission to MNH. This included any primary treatment received during the pre-hospital interval and the response to it, the nature of the provider/’patient/family member/doctor, place of treatment (traditional healers, pharmacy/nursing home, private hospital, primary health center), and form of treatment (antibiotic, analgesic, antacid, laxative, intravenous fluids, etc.) The events during the pre-hospital interval were identified and classified as related to the patient and family, to the medical personnel, or other reasons such as late referral, off-duty hours, public holidays, etc. The signs and symptoms at the presentation time were noted and a detailed history was recorded. The provisional diagnosis and clinical decision made by the attending healthcare professional at the time of admission to MNH were recorded.

### Statistical analysis

Data were entered in Microsoft Excel and analyzed using the SPSS software version 20. A *p* value < 0.05 was considered statistically significant. Collected data were summarized and explained using frequency distribution tables, a chi-square and Fisher-exact was performed to determine the factors and outcomes that may be related to the patients with a clinical diagnosis of surgical acute abdomen who presented late to the hospital.

## Results

### Demographic characteristics

A total of 170 patients with a clinical diagnosis of surgical acute abdomen participated in this study; of them, 115 (67.6%) were men and the highest percentage (35.5%) belonged to the age group of 21–40 years. The proportions of patients with primary education were greater (n = 75, 44.1%) than those at other educational levels. The majority of participants (n = 74, 43.5%) were self-employed, with only a few government employees (n = 9, 5.3%). Most patients (n = 97, 56.1%) who visited our hospital belonged to the low-income group. Table 1 provides detailed demographic characteristics of the patients.

**Table 1:** Social demographic characteristics of the surgical acute abdomen patients

Variable	Category	Frequency (n)	Percent (%)
Age years	0–20	39	22.9
	21–40	60	35.3
	41–60	50	29.4
	> 60	21	12.4
	Mean 37, SD 20, Max 85, Min 0.2		
Sex	Male	115	67.6
	Female	55	32.4
Religions	Christian	97	57.1
	Muslim	67	39.4
	Others	6	3.5
Level of education	Non-formal education	12	7.1
	Primary education	75	44.1
	Secondary education	54	31.8
	College/university	19	11.2
	None	10	5.9
Occupation	Government employed	9	5.3
	Private employed	19	11.2
	Self-employed	74	43.5
	Unemployed	68	40
Monthly income (TSH)	Low (< 300,000)	97	57.1
	Middle (300,000–900,000)	47	27.6
	High (900,000 and above)	8	4.7
	None	18	10.6

### Risk factors for late presentation of patients with acute surgical abdomen at MNH

This analysis supports that patients with surgical acute abdomen older than 60 (76.2%) years presented to the hospital later compared to those aged 0–40 years (*P* = 0.05). Similarly, males presented later than females (54.8% vs. 45.5%, *P* = 0.325). When we examined the influence of education on delayed presentation of patient with surgical acute abdomen, we found no statistically significant differences (*P* > 0.05). The majority of government officers (55.6%) did not arrive late, although there was no statistical significance compared to those in the private sector or who were self-employed (52.6% and 54.1%, respectively, *P* = 0.94). Moreover, a review of marital status (*P* = 0.03) revealed that the majority of widows, people living with their parents, and those from cohabiting family members (69.2%, 70.6%, and 88.8%, respectively) presented late to the wards. When comparing the availability of medical staff and time from initiation of pain to our surgery room, most of the cases from peripheral hospitals with less than 2 health care staff on duty presented late (52.7%) than patients from hospitals with more than 2 staff, although the difference was statistically insignificant (*P* = 0.751).

Table 2 provides a summary of the comparative features.

**Table 2:** Socio-demographic and health sector variables and their impact on the outcome for study participants

Variable	Early presentation (< 24 h)	Late presentation (> 24 h)	Total	*P* value
*Age (Years)*				
0–20	23 (59.0)	16 (41.0)	39	0.05
21–40	32 (53.3)	28 (46.7)	60	
41–60	22 (44.0)	28 (56.0)	50	
> 60	5 (23.0)	16 (76.2)	21	
Total	**82**	**88**	**170**	
*Sex*				
Males	52 (45.2)	63 (54.8)	115	0.325
Females	30 (54.5)	25 (45.5)	55	
Total	**82**	**88**	**170**	
*Level of education*				
Non formal	2 (16.7)	10 (83.3)	12	0.121
Primary	38 (50.7)	37 (49.3)	75	
Secondary	28 (51.9)	26 (48.1)	54	
College/university	11 (57.9)	8 (42.1)	19	
None	3 (30)	7 (70.0)	10	
Total	**82**	**88**	**170**	
*Employment status*				0.94
Government	5 (55.6)	4 (44.4)	9	
Private	9 (47.4)	10 (52.6)	19	
Self	34 (45.9)	40 (54.1)	74	
Unemployed	34 (50.0)	34 (50.0)	68	
Total	**82**	**88**	**170**	
*Marital status*				
Married	41 (48.2)	44 (51.8)	85	
Single	31 (67.4)	15 (32.6)	46	
Widow	4 (30.8)	9 (69.2)	13	
Cohabitating	1 (11.1)	8 (88.9)	9	
Living with parents	5 (29.4)	12 (70.6)	17	
*Religion*				
Christian	45 (46.4)	52 (53.6)	97	0.188
Muslims	36 (53.7)	31 (46.3)	67	
Others	1 (16.7)	5 (83.3)	6	
*Staff's*				
Adequacy				
Enough > 2	30 (50)	30 (50)	60	0.751
Less < 2	52 (47.3)	58 (52.7)	110	

Bold values are used to differentiate the total numbers from others

## Discussion

Surgical acute abdomen is a vaguely defined term referring to any condition causing abdominal pain of sudden onset and or short duration. Most causes are those within the abdomen. However, there are a few exceptions. It is a subjective unpleasant sensation felt in any part of the abdomen that may indicate a life-threatening intra-abdominal pathology requiring urgent surgical intervention [1, 3, 5, 16].

In a developing country like Tanzania, the practice of medicine, referral patterns, access to healthcare, and awareness of medical emergencies differ from those in the developed world, making it difficult to make a definitive diagnosis. However, early presentation facilitates appropriate management and a good outcome [2, 4]. Most studies from the developing world have focused on complications related to delayed diagnosis of specific abdominal problems like acute appendicitis. Only a few of them have assessed factors related to delay in presentation of patients with a surgically acute abdomen [5, 19].

The purpose of our study was to determine how socio-demographic and health-related factors contribute to a delayed presentation of surgical acute abdomen patients at MNH [6, 16]. Accordingly, we divided patients into two groups based on a random time of 24 h, representing early and late presentation, analyzed the reasons for the delay in diagnosis and treatment, and evaluated the prognosis for each group.

In this study, many patients with surgical acute abdomen arrived late at MNH because 75% of them were referred from the peripheral hospitals for special surgical intervention and other treatments. MNH is the first referral hospital in Tanzania with a high capacity for surgical intervention and can manage more common surgical cases, such as perforated appendicitis, intestinal obstruction, strangulated hernias, abdominal visceral injury, etc. Consequently, these patients are more likely to be referred to a tertiary hospital [8, 9, 13].

Inadequate health staff during the arrival of these patients at the peripheral hospitals, insufficient awareness of the disease and misdiagnosis, lack of awareness of medical facilities, social and cultural values were all associated with late presentation [7, 13, 19].

Moreover, private and self-employed patients showed delayed presentation for emergency abdominal surgery compared to government employees, which may be because the former have more freedom than the latter, and the government can support them (through health insurance services). Lack of health insurance and paying from their pockets leave the latter group of individuals waiting for a long time at their homes because they do not have enough money to go to hospitals [20, 21]. Self-employed individuals with surgical acute abdomen came late to MNH because it was difficult for them to leave their daily activities and go to the hospital. They were given a painkiller from the pharmacy and waited until their condition worsened, which resulted in a delay in appropriate treatment as well [10, 22]. Therefore, it is important to triage patients according to the surgical acute abdomen.

Upon analyzing the demographic parameters, we discovered that as the patients' age increased, they were more likely to arrive late for their urgent abdominal surgery. This was consistent with previous studies because older adults with chronic conditions faced several physical, social, and psychological challenges as a result required assistance to support their hospital visits, where they would otherwise be unable to be seen on time, and because they had numerous complaints, so their families did not listen to each of them [11, 23–26]. This was different from the younger age group because they were still under parental supervision. Of those patients, males showed more delay in presenting at MNH compared to females, and this sex disparity and its effect were documented in a prior cohort [9, 11]. The possible explanation is that males are affected by surgical acute abdomen more than females, therefore, upon diagnosis, they were classified as late, consistent with previous findings [9, 27, 28].

Patients from cohabiting families with surgical acute abdomen showed a delay in the presentation at MNH, which may be because Tanzanian cultural characteristics and norms do not favor this type of family, causing them to create dread and delay the surgical procedures more than patients with other marital statuses. In contrast, a prior survey found that cohabiting families were more susceptible to health problems because some countries embrace cohabitation as other families do [11, 29]. Widows present late for the surgical acute abdominal care at MNH because their lives are arduous, and they may have multiple children on their backs, making it difficult for them to reach a hospital in time [11, 29, 30] Educated groups, especially college graduates, were less prone to presenting late than those with informal education and uneducated individuals. Although a statistically insignificant difference, it was obvious that most uneducated individuals did not pay more attention to health issues unlike educated people [30].

This study has certain limitations that must be acknowledged. First, this was a single-center descriptive cross-sectional study, so the external validity of the results is restricted. However, MNH gets referrals from all across the nation daily, and the researchers took care to gather data from all patients who satisfied the inclusion criteria. There was a possibility of an inclusion bias, and the frequency of diagnoses among individuals referred is not likely representative of the nation as a whole.

In critically sick patients, relying on the informant's account may have resulted in incomplete data collection. This was minimized by obtaining a detailed patient or informant history and a thorough physical examination of the patients performed by the clinician. Our health system lacks access to essential aspects, such as insurance and medical checkups, that may have altered the presentation timing. Therefore, further comprehensive prospective multicenter studies are required to analyze the existing situation and provide a solution to reduce the delay in presentation and high fatality rate among these individuals.

Our advice to the Ministries of Health is to create special programs that can be broadcasted on social media, TV, radio, and medical camps, creating a short course for medical professionals (doctors, nurses, radiologists, etc.) on handling emergency cases on time, and educating the community about the risks and benefits of visiting the hospital as soon as they feel symptoms of the illness (avoid using a painkiller before seeing the doctor, older individuals, workers in the private sectors, etc.). To minimize the mortality rate caused by poor health infrastructure, developed countries should support urban and rural health sectors in all underdeveloped countries.

## Conclusion

Overall, the mortality rate of patients with surgical acute abdomen often increases with delayed presentation. Informal education, cohabiting families, self and private employment, advancing age, insufficient health staff on duty, and poor infrastructure were identified as factors that may contribute to the late presentation of these patients at MNH in Tanzania. Further research is needed to improve the quality of care, which should focus primarily on determining why these variations arise, identifying other possible factors, and ways to reduce death rates.

## Data Availability

The datasets used and/or analysed during the current study available from the corresponding author on reasonable request.

## Abbreviations

WHO: World Health Organization
MUHAS: Muhimbili University of Health and Allied Science
MNH: Muhimbili National Hospital
SPSS: Statistical package for social sciences
LMIC: Low and middle-income countries
PI: Principle investigator
RA: Research assistant
PUD: Peptic ulcer disease
ED: Emergency department
GSD: General surgery department
